# Targeting neuroinflammation: itaconate and mesaconate as therapeutic strategies against H7N7 influenza-associated CNS damage

**DOI:** 10.3389/fimmu.2026.1776302

**Published:** 2026-03-03

**Authors:** Melanie Ohm, Wei He, Dunja Bruder, Karsten Hiller, Martin Korte, Shirin Hosseini

**Affiliations:** 1 Department of Cellular Neurobiology, Zoological Institute, Technische Universität (TU) Braunschweig, Braunschweig, Germany; 2 Department of Bioinformatics and Biochemistry, Braunschweig Integrated Centre of Systems Biology (BRICS), TU Braunschweig, Braunschweig, Germany; 3 Research Group Infection Immunology, Institute of Medical Microbiology and Hospital Hygiene, Otto-von-Guericke-University Magdeburg, Magdeburg, Germany; 4 Research Group Immune Regulation, Helmholtz Centre for Infection Research, Braunschweig, Germany; 5 Neuroinflammation and Neurodegeneration Group, Helmholtz Centre for Infection Research, Braunschweig, Germany

**Keywords:** hippocampus, influenza A virus infection, metabolites, microglia, synaptic plasticity

## Abstract

**Introduction:**

Influenza A virus (IAV) infection is primarily associated with respiratory disease; however, accumulating evidence indicates that neurotropic strains can induce central nervous system (CNS) inflammation and contribute to persistent neurological dysfunction. Aberrant immune activation is thought to play a critical role in these outcomes, yet therapeutic approaches that effectively attenuate neuroinflammation while preserving antiviral immunity remain limited. Immunometabolic regulators, including the endogenous metabolite itaconate, have recently emerged as key modulators of innate immune responses, although their contribution to virus-induced CNS pathology remains incompletely understood.

**Methods:**

In the present study, we investigated whether systemic administration of itaconate or its structural isomer mesaconate modulates neuroinflammatory responses and hippocampal synaptic integrity during infection with the neurotropic IAV strain rSC35M (mouse-adapted A/Seal/Mass/ 1/80, H7N7). Using a murine model, treatment was initiated at the onset of clinical symptoms, and both peripheral and central immune responses were assessed at the peak of disease.

**Results and discussion:**

Neither itaconate nor mesaconate significantly altered overall disease severity, as assessed by body weight loss, although mesaconate attenuated infection-associated hypothermia. Pulmonary inflammatory responses were largely unaffected by treatment; in contrast, mesaconate selectively reduced IL-1β levels in the brain. At the cellular level, H7N7 infection induced pronounced microglial activation within hippocampal subregions, characterized by increased cell density and soma volume, altered process complexity, and enhanced engulfment of postsynaptic material. These infection-induced microglial alterations were partially prevented by mesaconate treatment and largely abrogated by itaconate treatment. Notably, attenuation of microglial density and reactivity during the acute phase was associated with long-term preservation of hippocampal synaptic plasticity. Collectively, these findings indicate that therapeutic administration of itaconate and mesaconate, potentially through distinct mechanisms, can modulate microglia-driven synaptic pathology during neurotropic IAV infection. Targeting immunometabolic pathways may therefore represent a promising strategy to prevent persistent neurological sequelae associated with viral disease.

## 1 Introduction

Influenza A viruses (IAVs) are enveloped, negative-sense RNA viruses of the *Orthomyxoviridae* family that continue to pose a major public health burden globally, causing recurrent seasonal epidemics and occasional pandemics with substantial morbidity and mortality. The segmented genome of IAV facilitates genetic variation through antigenic drift and shift, enabling the frequent emergence of novel strains with altered antigenicity, host range, and pathogenicity (1, 2). These evolutionary mechanisms underlie the persistence of IAV as a significant threat to human health and complicate both disease control and vaccine design.

Although the primary tropism of IAV is the respiratory epithelium, a growing body of evidence indicates that IAV infection can affect the central nervous system (CNS) through both direct and indirect mechanisms (3). In humans, severe influenza infection has been associated with neurological complications, including acute influenza-associated encephalopathy and encephalitis, characterized by altered consciousness, seizures, cerebral edema, and, in some cases, long-term neurocognitive consequences (4, 5). Highly pathogenic and neurotropic strains such as H5N1 and H7N7 can breach or bypass the blood-brain barrier (BBB), infect neural cells, and induce local inflammatory responses associated with neuronal dysfunction and neuropathological changes (6–8). Indirectly, even non-neurotropic seasonal IAV strains, including H1N1 and H3N2, can trigger systemic inflammatory cascades that influence CNS function. Peripheral infection induces robust cytokine and chemokine production that may compromise BBB integrity, activate resident CNS immune cells such as microglia and astrocytes, and propagate neuroinflammatory signaling into the brain (7, 9, 10). Both systemic and CNS-specific immune responses to IAV infection have been associated with alterations in hippocampal structure, increased neuroinflammation, and cognitive impairment in animal models (7, 11).

Given the limited regenerative capacity of the adult CNS, disruption of neural homeostasis during influenza infection may precipitate prolonged or maladaptive neurological sequelae. Accordingly, there is significant interest in identifying interventions that can attenuate both peripheral and CNS inflammatory responses without impairing antiviral defense.

Recent investigations have identified endogenous immunometabolic regulators as key modulators of host responses during viral infection. Itaconate, generated by aconitate decarboxylase 1 (ACOD1/IRG1) in activated macrophages, along with its derivatives dimethyl itaconate (DMI) and 4-octyl itaconate (4-OI), exerts broad anti-inflammatory and immunomodulatory effects (12, 13). In models of respiratory viral infection, exogenous itaconate, DMI, and 4-OI suppress exaggerated type I interferon and pro-inflammatory cytokine signaling in human cells and *ex vivo* human lung tissue, while modulating reactive oxygen species and NRF2-dependent antioxidant pathways (14, 15). *In vivo*, DMI reduces pulmonary inflammation and improves survival in IAV-infected mice, whereas loss of *Acod1* exacerbates disease severity, demonstrating a protective function for endogenous itaconate (15). Notably, these compounds attenuate cytokine and interferon responses without enhancing viral replication, and 4-OI can additionally restrict viral ribonucleoprotein nuclear export, contributing to antiviral activity (16, 17).

He et al. further reported that lipopolysaccharide (LPS) stimulation of macrophages induces the production of both itaconate and mesaconate, a structural isomer differing only in the position of a carbon-carbon double bond. Both metabolites exhibit anti-inflammatory activity, as treatment with either itaconate or mesaconate reduces pro-inflammatory cytokine production in activated peripheral macrophages (18). In addition, a study by Ohm et al. demonstrated the immunomodulatory and therapeutic potential of itaconate and mesaconate in LPS-induced neuroinflammatory processes *in vivo*. Their findings showed that both metabolites attenuate LPS-induced neuroinflammation, as evidenced by reduced levels of inflammatory mediators, decreased microglial reactivity, and preservation of synaptic plasticity – the cellular correlate of learning and memory in the brain (19).

Collectively, these findings highlight itaconate- and mesaconate-derived metabolites as potent host-directed immunomodulators capable of limiting pathogenic inflammation during influenza virus infection.

To determine whether endogenous itaconate and its structural isomer mesaconate modulate neuroinflammatory responses during IAV infection, we employed a murine model using the neurotropic rSC35M mouse-adapted A/Seal/Mass/1/80 (H7N7) strain. Mice received systemic treatment via intraperitoneal injections of itaconate or mesaconate starting at the onset of clinical symptomatology (3 days post-infection). Brains were collected and analyzed at 8 days post-infection, corresponding to the peak of disease (7). This experimental design enabled evaluation of whether metabolic supplementation with these endogenous immunomodulators attenuates IAV-induced neuroinflammation and contributes to the preservation of neural homeostasis.

## 2 Materials and methods

### 2.1 Animals

To investigate the anti-inflammatory effects of itaconate and mesaconate during IAV infection, two-month-old female C57BL/6J inbred mice (Janvier, France) were used. Animals were housed under specific pathogen-free conditions at the Helmholtz Centre for Infection Research (HZI), Braunschweig, in accordance with German animal welfare regulations. Mice were maintained on a 12-h light/dark cycle with *ad libitum* access to food and water and were group-housed with six animals per cage under controlled environmental conditions. All animal procedures were conducted in compliance with national and institutional ethical guidelines. Experimental protocols were approved by the institutional animal welfare committees of the HZI and TU Braunschweig, as well as by the Lower Saxony State Office for Consumer Protection and Food Safety (LAVES), Oldenburg (permit number: 33.19-42502-04-18/2968), in accordance with the German Animal Welfare Act (Tierschutzgesetz, revised 18 May 2006; BGBl. I S. 1206, 1313).

### 2.2 Itaconate and mesaconate treatment during influenza A virus infection

Mice were anesthetized by intraperitoneal injection of a ketamine-xylazine mixture composed of 85% NaCl (0.9%), 10% ketamine (100 mg/mL), and 5% xylazine (20 mg/mL), delivered at 10 mL/kg body weight. After achieving adequate anesthesia, animals were intranasally inoculated with a sublethal dose of 10 focus-forming units (FFU) of the mouse-adapted influenza A virus A/Seal/Mass/1/80 (H7N7) (rSC35M) suspended in 20 µL of sterile 1× PBS, following established protocols (7). Control mice received an identical intranasal volume of sterile 1× PBS.

Body weight, body temperature, and physical condition – including spontaneous activity, posture, fur appearance, and response to provocation – were monitored daily throughout the acute infection phase (up to 8 days post-infection, dpi). Percent body weight loss was calculated relative to baseline weight on day 0. Animals exhibiting >30% body weight loss or signs of severe distress were humanely euthanized according to ethical guidelines.

Beginning at 3 dpi, corresponding to the onset of clinical symptoms, mice received intraperitoneal injections of itaconate or mesaconate at a dose of 250 mg/kg body weight. Treatments were administered at 3, 5, and 7 dpi, following the dosing regimen described by previous studies to elicit immunoregulatory effects (18, 19). Control animals received equivalent volumes of 1× PBS, the vehicle used for metabolite preparation. Prior to each injection, mice were examined for any abnormalities that could interfere with treatment administration or subsequent analyses. Daily measurements of body weight and general health parameters were used to adjust the injection volume to ensure accurate dosing.

### 2.3 Enzyme-linked immunosorbent assays

Cytokine quantification was performed using ELISA. At 8 dpi, mice were deeply anesthetized and euthanized with CO_2_ using a gradual-fill method with a displacement rate of 30-70% of the chamber volume per minute, followed by decapitation. Lungs and brains were rapidly extracted, snap-frozen in liquid nitrogen, and stored at -70 °C until further processing. Here, brains were collected without transcardial perfusion, as this approach has previously been shown to yield accurate measurements of brain cytokine levels without introducing bias from circulating blood components (20); therefore, this time-consuming step was omitted. Frozen lung and brain tissues were homogenized in 1000 µL and 500 µL of STKM lysis buffer, respectively (250 mM sucrose, 50 mM Tris-HCl, 25 mM KCl, 5 mM MgCl_2_), supplemented with a protease inhibitor cocktail (Roche cOmplete™), using an Eppendorf-fitting pestle. Homogenates were centrifuged at 13,000 × g for 10 min at 4 °C. Final supernatants were stored at -70 °C until ELISA analysis.

Pro-inflammatory cytokine concentrations (TNF-α: Cat. # DY410, assay range: 31.2–2000 pg/mL; IL-6: Cat. # DY406, assay range: 15.6–1000 pg/mL; IL-1β: Cat. # DY401, assay range: 15.6–1000 pg/mL and IFN-γ: Cat. # DY485, assay range: 31.2–2000 pg/mL) were quantified using DuoSet ELISA kits (R&D Systems) according to the manufacturer’s instructions. Briefly, 96-well plates were coated overnight with the appropriate capture antibodies, washed, and incubated for 2 h with standards and diluted samples (1:2). Following additional washing steps, detection antibodies were applied for 2 h, followed by incubation with substrate solution for 20 min in the dark. The reaction was terminated with stop solution, and absorbance was measured at 450 nm using an Epoch microplate reader (BioTek). Data acquisition and analysis were performed using Gen5 software. To normalize cytokine concentrations to total protein content, protein levels were determined using a Bradford assay. Homogenates were diluted 1:200 in STKM buffer, and absorbance was measured at 595 nm using the same microplate reader.

### 2.4 Immunohistochemistry

Immunohistochemistry was performed to identify microglial cells in brain tissue. Mice were deeply anesthetized and euthanized by CO_2_ inhalation using a gradual-fill method (30-70% chamber volume per minute), followed by decapitation. Brains were carefully removed, fixed in 10 mL of 4% paraformaldehyde (PFA) for 24 h at 4 °C, and subsequently cryoprotected in 30% sucrose in 1× PBS until the tissue sank. Brains were then embedded in Tissue-Tek^®^ O.C.T.™ compound (A. Hartenstein Laborversand), frozen at -70 °C, and coronally sectioned at a thickness of 25 µm using a Leica 2800E Frigocut cryostat.

Five consecutive sections per mouse were transferred to 24-well plates for free-floating immunohistochemistry. Sections were washed twice in 1× PBS for 2 min each, followed by three washes in 0.1% Triton X-100 for 5 min each, with constant agitation on a shaker. Blocking was performed for 1 h at room temperature (RT) using 1× PBS containing 0.3% Triton X-100, 5% goat serum, 5% donkey serum, and 5% bovine serum albumin (BSA). Sections were then incubated overnight at 4 °C with primary antibodies diluted in blocking solution, including rabbit anti-IBA-1 (1:1,000; Synaptic Systems, RRID: AB_10641962), rat anti-mouse CD107a/LAMP-1 (1:500; BD Pharmingen™, RRID: AB_2134499), and chicken anti-Homer-1 (1:500; Synaptic Systems, RRID: AB_2631222). The following day, sections were equilibrated for 30 min at RT on a shaker and washed three times in 1× PBS for 10 min each. Sections were then incubated for 2 h at RT in the dark with fluorophore-conjugated secondary antibodies (1:500), diluted in 0.05% Triton X-100 in 1× PBS: Cy™3 goat anti-rabbit IgG (H+L) (RRID: AB_2338006), Cy™5 goat anti-rat IgG (H+L) (RRID: AB_2338264), and Alexa Fluor^®^ 488 donkey anti-chicken IgY (H+L) (RRID: AB_2340375). After incubation, sections were washed three times in 1× PBS (10 min each), incubated with DAPI (1:1,000; Sigma-Aldrich) for 5 min, and washed an additional six times in 1× PBS (5 min each). Finally, stained sections were mounted onto glass slides using Fluorogel mounting medium (Electron Microscopy Sciences, Hatfield, PA).

### 2.5 Imaging and image analysis

#### 2.5.1 Imaging and quantification of IBA-1^+^ cell density and fluorescence intensity

Immunohistochemically stained sections were imaged using an Apotome microscope (Imager.M2 AXIO, ZEISS) equipped with a ×20 objective (NA 0.8). Only the DAPI and Cy™3 channels were acquired for the detection of IBA-1-positive cells. Z-stacks were collected with a step size of 1 µm from four to five hippocampal sections per animal. For each section, the CA1 and dentate gyrus subregions of the hippocampus were imaged to assess microglial cell density. Image analysis was performed in a blinded manner using Fiji software (BioVoxxel). From each Z-stack, eight optical sections from the central portion of the stack were selected and projected into a two-dimensional image using the “Z Project” function with the “maximum intensity” setting. The Cy™3 and DAPI channels were then merged using the “Color” and “Merge Channels” tools. IBA-1^+^ microglial cells within each image were manually counted using the “Multiple Points” tool. Cell density was calculated as the number of cells per mm² using Microsoft Excel. Fluorescence intensity of IBA-1 immunoreactivity was also quantified. To identify positive signals, the integrated density of the selected region of interest (whole frame) was measured along with three background regions lacking specific staining. Background values were averaged and subtracted from the total integrated density to obtain the corrected fluorescence signal. All data were normalized within each staining to the mean value of the corresponding control group (PBS-PBS).

#### 2.5.2 Single-cell imaging and analysis of microglia

Individual, randomly selected microglial cells were imaged from triple-stained sections (IBA-1/LAMP-1/Homer-1) (21). Z-stack images were acquired using a confocal laser scanning microscope (cLSM; Olympus) with a ×40 UPLFLN oil-immersion objective (NA 1.30) and a ×6 digital zoom. Optical sections were collected at 0.35-µm intervals, resulting in a final pixel size of 0.103 × 0.103 µm. For each animal, Z-stacks of three randomly selected individual microglial cells from the CA1 and dentate gyrus subregions were acquired from three independent sections. Prior to analysis, images were subjected to blind three-dimensional deconvolution using AutoQuantX (Adobe Systems GmbH).

Three-dimensional reconstruction and quantitative analysis were performed using IMARIS^®^ software (Bitplane). Microglial cell surfaces were generated based on IBA-1 immunoreactivity (surface detail: 0.2 µm). Within the reconstructed microglial surfaces, LAMP-1-positive signals were masked to generate three-dimensional surface models of LAMP-1-positive vesicles (surface detail: 0.2 µm). Homer-1 puncta located within LAMP-1-positive vesicles were identified and quantified using the spot detection function (spot diameter: 0.5 µm).

Microglial morphology was further assessed by masking the IBA-1 signal into the reconstructed IBA-1 surface to generate filament models for analysis of branching complexity. Filament reconstruction parameters included a largest diameter of 5 µm, a minimum diameter of 0.3 µm, and a sphere region diameter of 15 µm. This approach was also used to quantify microglial soma size. Quantified parameters included IBA-1-positive volume (µm³), LAMP-1 volume within IBA-1-positive structures (µm³), the number of Homer-1 puncta within LAMP-1-positive compartments, Sholl analysis of microglial processes, and microglial soma volume (µm³). All measurements were exported to Microsoft Excel for further analysis. Data were normalized within each staining to the mean value of the corresponding PBS-PBS control group.

### 2.6 Electrophysiological experiments

At 30 dpi, brains were rapidly isolated and immediately transferred to ice-cold, carbogenated (95% O_2_ and 5% CO_2_) artificial cerebrospinal fluid (ACSF) containing (in mM): 124 NaCl, 4.9 KCl, 1.2 KH_2_PO_4_, 2.0 MgSO_4_, 2.0 CaCl_2_, 24.6 NaHCO_3_, and 10 D-glucose (pH 7.4). Transverse hippocampal slices (400 µm thickness) were prepared using a manual tissue chopper. Slices were transferred to an interface recording chamber (Scientific System Design) and maintained at 32 °C under a constant flow (0.5 mL/min) of carbogenated ACSF for at least 2 h prior to electrophysiological recordings.

Field excitatory postsynaptic potentials (fEPSPs) were recorded from the stratum radiatum of the CA1 hippocampal subregion. Synaptic responses were evoked by electrical stimulation of the Schaffer collateral pathway connecting CA3 to CA1 using a monopolar, lacquer-coated stainless-steel stimulating electrode (5 MΩ; AM Systems). Recording electrodes (5 MΩ; AM Systems) were positioned in the CA1 apical dendritic layer at least 20 µm from the stratum pyramidale. Signals were amplified using a differential amplifier (Model 1700, AM Systems) and digitized with a CED 1401 analog-to-digital converter (Cambridge Electronic Design).

Basal synaptic transmission was assessed by generating input-output curves relating afferent stimulation intensity to the fEPSP slope, measured as the initial rising phase of the fEPSP. Stimulus intensity was adjusted to evoke responses corresponding to 40% of the maximal fEPSP slope and was kept constant throughout the experiment. After establishing a stable baseline for at least 20 min, long-term potentiation (LTP) was induced using theta-burst stimulation (TBS), consisting of four pulses at 100 Hz per burst, repeated 10 times at 200-ms intervals. This stimulation protocol was applied three times with an inter-train interval of 10 s. fEPSP slopes were recorded for 60 min following TBS and normalized to baseline values. Data acquisition and offline analysis were performed using IntraCell software (version 1.5; LIN, Magdeburg, 2000), as previously described (7, 19).

### 2.7 Statistical analysis

Data are presented as mean ± SEM and were analyzed and graphically displayed using GraphPad Prism 9 (GraphPad Software, Inc., USA). Differences between experimental groups were analyzed using two-way analysis of variance (ANOVA), with infection status and metabolite treatment as independent factors. Ordinary or repeated-measures two-way ANOVA was applied as appropriate, depending on the experimental design. When main effects or interactions were detected, Fisher’s least significant difference (LSD) test was used for *post hoc* multiple comparisons. Statistical significance was defined as p < 0.05. Sample sizes were determined *a priori* using G*Power software (version 3.1.9.4; Heinrich Heine University Düsseldorf, Germany). The number of animals (N) and samples (n) for each experimental group are indicated in the corresponding figure legends. For cytokine measurements, technical replicates were averaged, and data are presented with the individual mouse defined as the experimental unit. All statistical analyses for these experiments were therefore performed at the animal level. In contrast, for microglial quantification and single-cell confocal analyses, data are presented at the level of individual regions of interest (ROIs) and individual microglial cells. This analytical approach was intentionally selected to account for the pronounced spatial, morphological, and phenotypic heterogeneity of microglia within the hippocampus, as well as the high sensitivity and resolution of image-based analyses. For electrophysiological experiments conducted *ex vivo*, individual acute hippocampal slices were considered the experimental unit, as cellular responses in slice preparations are highly dependent on *ex vivo* experimental conditions.

All data acquisition and analyses were performed in a strictly blinded manner.

## 3 Results

### 3.1 Therapeutic administration of itaconate and mesaconate in H7N7 IAV infected mice

Based on our previous findings showing that itaconate and mesaconate attenuate LPS-induced neuroinflammation – characterized by reduced expression of inflammatory mediators, decreased microglial reactivity, and preservation of synaptic plasticity (19) – we next investigated whether these metabolites exert therapeutic effects on neuroinflammation induced by H7N7 IAV infection (7, 21).

**Figure 1:**
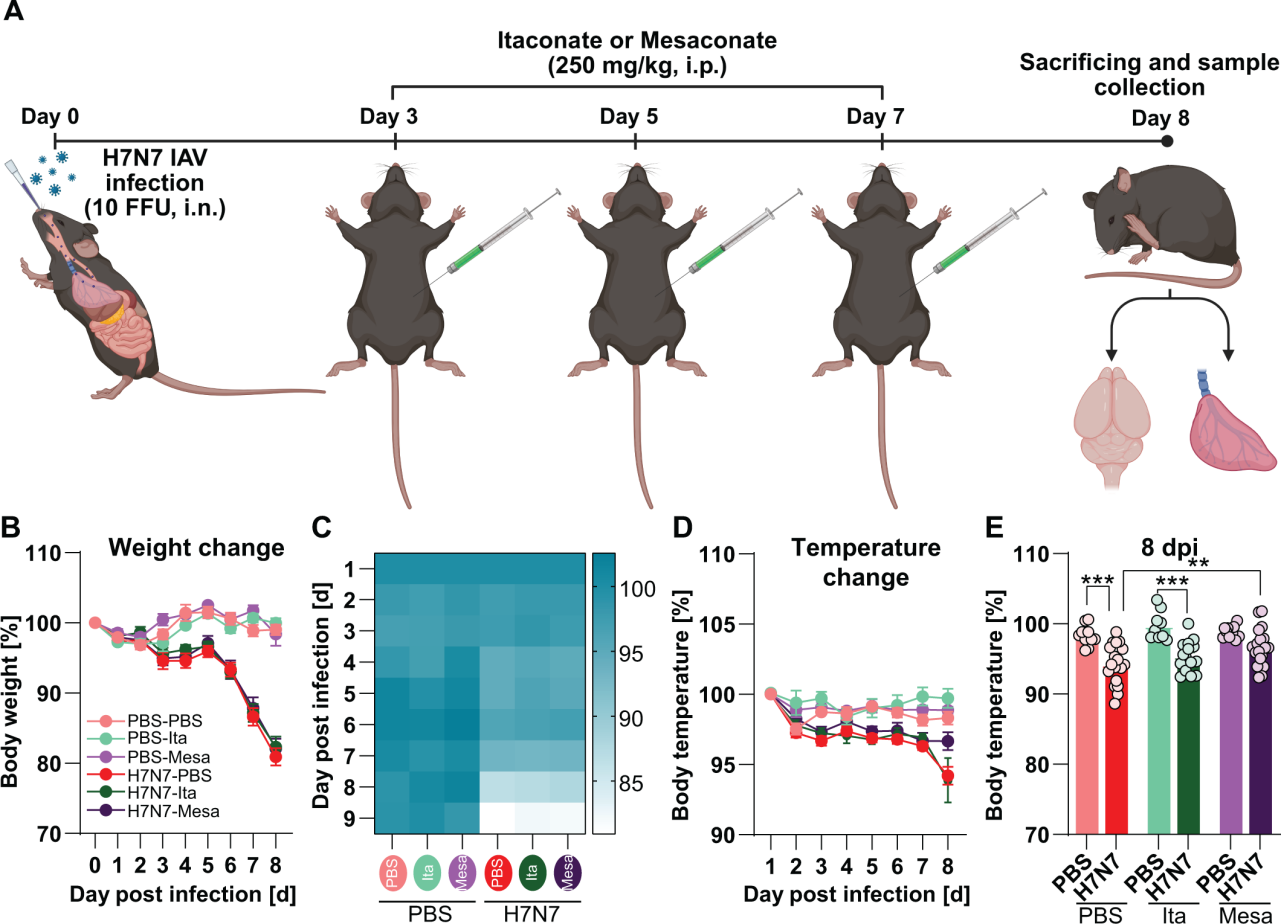
Effects of itaconate and mesaconate treatment on disease severity during acute H7N7 infection. **(A)** Experimental design (*Created in BioRender*). Two-month-old female mice were intranasally inoculated with PBS or infected with H7N7 IAV and treated intraperitoneally with PBS, itaconate (Ita), or mesaconate (Mesa; 250 mg/kg) on days 3, 5, and 7 post-infection. Animals were sacrificed at 8 dpi. **(B, C)** Body weight over time **(B)** and cumulative weight loss **(C)**. PBS-inoculated control mice maintained stable body weight, whereas H7N7-infected mice exhibited progressive weight loss independent of treatment. **(D)** Daily body temperature measurements showing infection-induced hypothermia in H7N7-infected mice. **(E)** Body temperature at 8 dpi. Hypothermia was observed in PBS- and itaconate-treated infected mice but was attenuated in mesaconate-treated infected mice. Data are presented as mean ± SEM. Statistical analysis was performed using two-way repeated-measures ANOVA followed by Fisher’s LSD *post hoc* test (**p < 0.01 and ***p < 0.001). Group sizes ranged from N = 15–19 mice per group.

Two-month-old female wild-type mice were intranasally infected with H7N7, while control animals received PBS. On days 3, 5, and 7 post infection (dpi), infected and control mice were treated intraperitoneally with either itaconate or mesaconate (250 mg/kg body weight) or PBS (18). This experimental design resulted in six treatment groups: PBS-PBS, PBS-Ita, PBS-Mesa, H7N7-PBS, H7N7-Ita, and H7N7-Mesa (**Figure 1A**). Body weight and body temperature were monitored daily until sacrifice at 8 dpi, corresponding to the peak of infection and maximal disease severity.

Body weight remained stable in all PBS-inoculated control groups, independent of metabolite treatment (**Figures 1B, C**). In contrast, H7N7-infected mice exhibited a progressive loss of body weight beginning at 3 dpi, which became more pronounced between 5 and 8 dpi, irrespective of treatment (**Figures 1B, C**). Neither itaconate nor mesaconate treatment prevented infection-associated weight loss (two-way repeated-measures ANOVA, main effect of infection: *F*(5, 95) = 26.44, *p* < 0.001).

Body temperature remained stable in PBS-inoculated control mice treated with PBS or metabolites. In contrast, H7N7-infected mice displayed a decline in body temperature starting at 3 dpi, with a marked reduction observed between 6 and 8 dpi (two-way repeated-measures ANOVA: *F*(5, 95) = 13.41, *p* < 0.001; **Figure 1D**). At 8 dpi, body temperature was significantly reduced in infected mice treated with PBS or itaconate compared with their respective control groups (PBS-PBS vs. H7N7-PBS, *p* = 0.003; PBS-Ita vs. H7N7-Ita, *p* = 0.001). In contrast, no significant reduction in body temperature was observed in mesaconate-treated infected mice (**Figure 1E**). Moreover, mesaconate-treated infected mice exhibited significantly higher body temperatures than infected mice treated with PBS or itaconate (H7N7-PBS vs. H7N7-Mesa, *p* = 0.03; H7N7-Ita vs. H7N7-Mesa, *p* = 0.02; **Figure 1E**).

Collectively, these data indicate that treatment with itaconate or mesaconate does not substantially alter general disease severity, as assessed by body weight loss, during acute H7N7 IAV infection. However, mesaconate treatment attenuated infection-induced hypothermia at 8 dpi, suggesting a selective beneficial effect on sickness-associated physiological responses during the peak of influenza infection.

### 3.2 Limited impact of itaconate and mesaconate on H7N7 IAV-induced inflammatory mediator production in the lung and brain at 8 dpi

Influenza infection is known to induce robust inflammatory responses and tissue damage, particularly in the lung as the primary target organ of respiratory viruses (22). In addition, both neurotropic and non-neurotropic IAV strains have been shown to trigger the release of inflammatory mediators in the brain during the acute phase of infection, contributing to virus-induced neuroinflammation (7, 9, 10).

**Figure 2:**
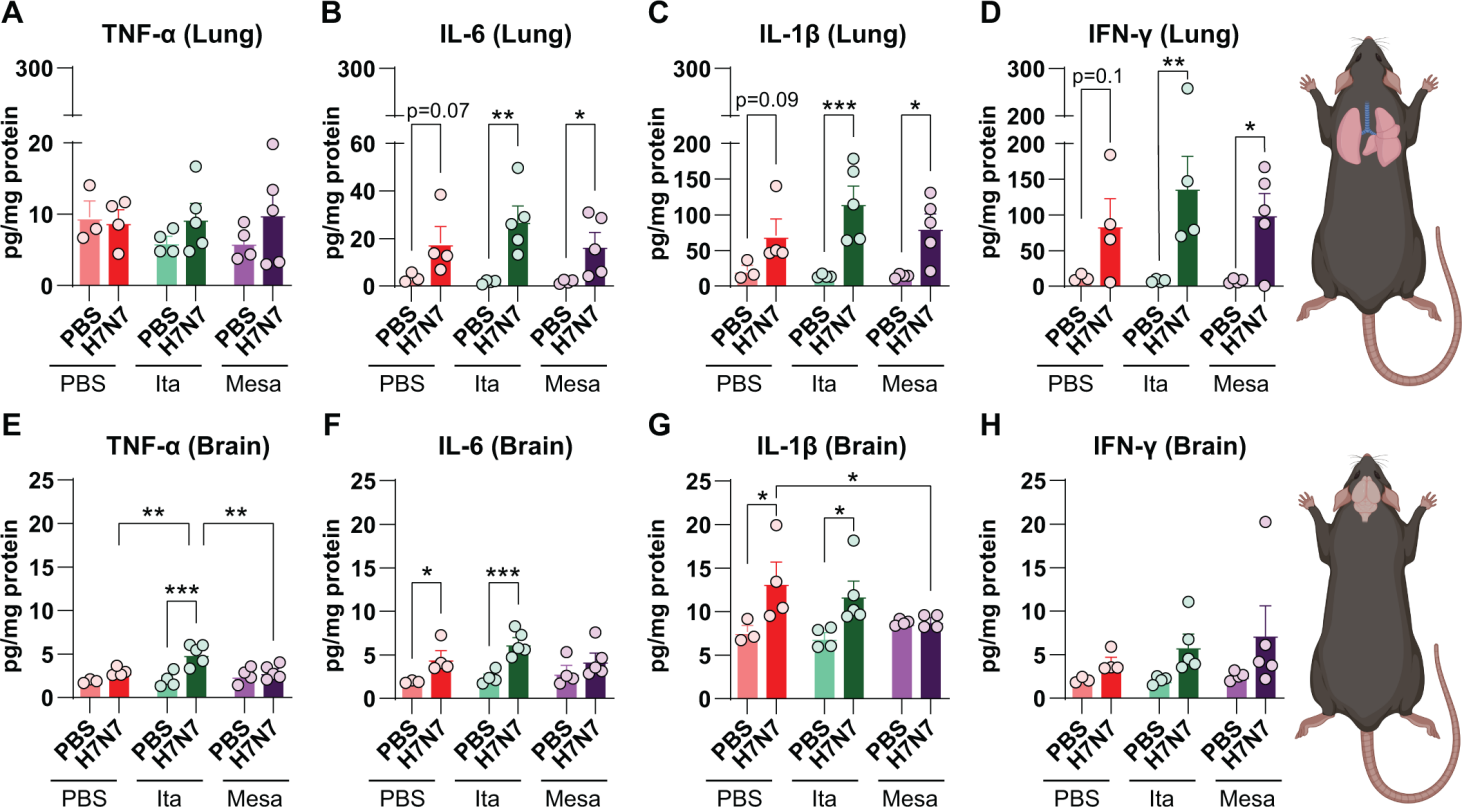
Limited effects of itaconate and mesaconate on H7N7-induced inflammatory mediator production in lung and brain at 8 dpi. Pro-inflammatory cytokine levels were quantified by ELISA in lung **(A–D)** and brain **(E–H)** tissues from PBS-inoculated control and H7N7-infected mice treated with PBS, itaconate, or mesaconate at 8 dpi. **(A–D)** H7N7 infection induced robust pulmonary increases in IL-6 **(B)**, IL-1β **(C)**, and IFN-γ **(D)**, which were not attenuated by either metabolite treatment. **(E–H)** In the brain, H7N7 infection increased IL-6 **(F)** and IL-1β **(G)** levels, with itaconate treatment further enhancing TNF-α production **(E)**, whereas mesaconate treatment was associated with reduced IL-1β **(G)** and attenuated neuroinflammatory responses. Data are presented as mean ± SEM. Statistical analysis was performed using two-way ANOVA followed by Fisher’s LSD *post hoc* test (*p < 0.05, **p < 0.01 and ***p < 0.001). Group sizes ranged from N = 3–5 mice per group.

To determine whether itaconate or mesaconate modulate inflammatory responses following IAV infection, pro-inflammatory cytokine concentrations were quantified in lung and brain tissues by ELISA (**Figure 2**).

Analysis of pulmonary cytokines revealed that H7N7-infected mice treated with PBS exhibited increased concentrations of IL-6, IL-1β, and IFN-γ compared with PBS-inoculated control mice (**Figures 2B–D**). Two-way ANOVA demonstrated a significant main effect of infection for IL-6 (*F*(1, 19) = 19.76, *p* = 0.0003), IL-1β (*F*(1, 19) = 24.52, *p* < 0.0001), and IFN-γ (*F*(1, 18) = 18.72, *p* = 0.0004). However, *post hoc* multiple-comparison analyses did not identify significant differences between individual PBS-treated infected and control groups. In contrast, H7N7-infected mice treated with either itaconate or mesaconate exhibited significantly elevated pulmonary cytokine levels relative to their respective non-infected control groups. Specifically, in itaconate-treated mice, H7N7 infection significantly increased IL-6 (*p* = 0.001), IL-1β (*p* = 0.0005), and IFN-γ (*p* = 0.003) concentrations (**Figures 2B–D**). Similarly, mesaconate-treated infected mice showed significantly higher levels of IL-6 (*p* = 0.03), IL-1β (*p* = 0.01), and IFN-γ (*p* = 0.02) compared with the corresponding PBS-mesaconate control group (**Figures 2B–D**). No significant differences in TNF-α concentrations were detected in lung tissue between infected and control mice, irrespective of treatment (two-way ANOVA, *F*(1, 19) = 1.51, *p* = 0.23; **Figure 2A**).

Whole brain hemispheres were homogenized to assess global neuroinflammatory responses to IAV infection and the potential broad anti-inflammatory effects of systemic itaconate and mesaconate administration by measuring pro-inflammatory cytokine concentrations in brain tissue from all experimental groups (**Figures 2E–H**). H7N7-infected mice treated with PBS exhibited significantly increased brain levels of IL-6 (*p* = 0.02) and IL-1β (*p* = 0.01) compared with PBS-inoculated control mice. Two-way ANOVA confirmed a significant main effect of infection for IL-6 (*F*(1, 19) = 19.35, *p* = 0.0003) and IL-1β (*F*(1, 18) = 10.37, *p* = 0.004) (**Figures 2F, G**).

Infected mice receiving itaconate displayed a more pronounced neuroinflammatory response. Relative to the corresponding itaconate-treated control group, H7N7 infection resulted in significantly elevated concentrations of TNF-α (*p* < 0.001), IL-6 (*p* = 0.0009), and IL-1β (*p* = 0.01) in the brain (**Figures 2E–G**). Notably, TNF-α levels in the brains of H7N7-infected itaconate-treated mice were significantly higher than those observed in infected mice treated with PBS or mesaconate (both *p* = 0.001; two-way ANOVA, *F*(1, 19) = 17.77, *p* = 0.0005; **Figure 2E**).

In contrast, mesaconate treatment was associated with attenuated neuroinflammatory responses. Brain IL-6 levels did not differ significantly between H7N7-infected mesaconate-treated mice and their corresponding control group, and IL-1β levels were significantly lower compared with infected PBS-treated mice (*p* = 0.03; **Figure 2G**).

For IFN-γ, two-way ANOVA revealed a significant main effect of infection (*F*(1, 19) = 5.52, *p* = 0.02). However, *post hoc* analyses did not detect statistically significant differences between individual infected and control groups. Nevertheless, a consistent trend toward increased IFN-γ levels was observed across all infected groups, independent of treatment (**Figure 2H**).

Collectively, H7N7 IAV infection increased the production of inflammatory cytokines in both lung and brain tissues. Treatment with itaconate or mesaconate did not attenuate pulmonary cytokine responses and was instead associated with enhanced inflammatory signaling following infection. In contrast, mesaconate treatment selectively reduced brain IL-1β levels and did not result in a significant increase in IL-6, suggesting a modest neuroprotective effect that may influence the severity of infection-associated neuroinflammatory outcomes.

### 3.3 Metabolite treatment modulates H7N7 IAV-induced microglial density and reactivity in the hippocampus

**Figure 3:**
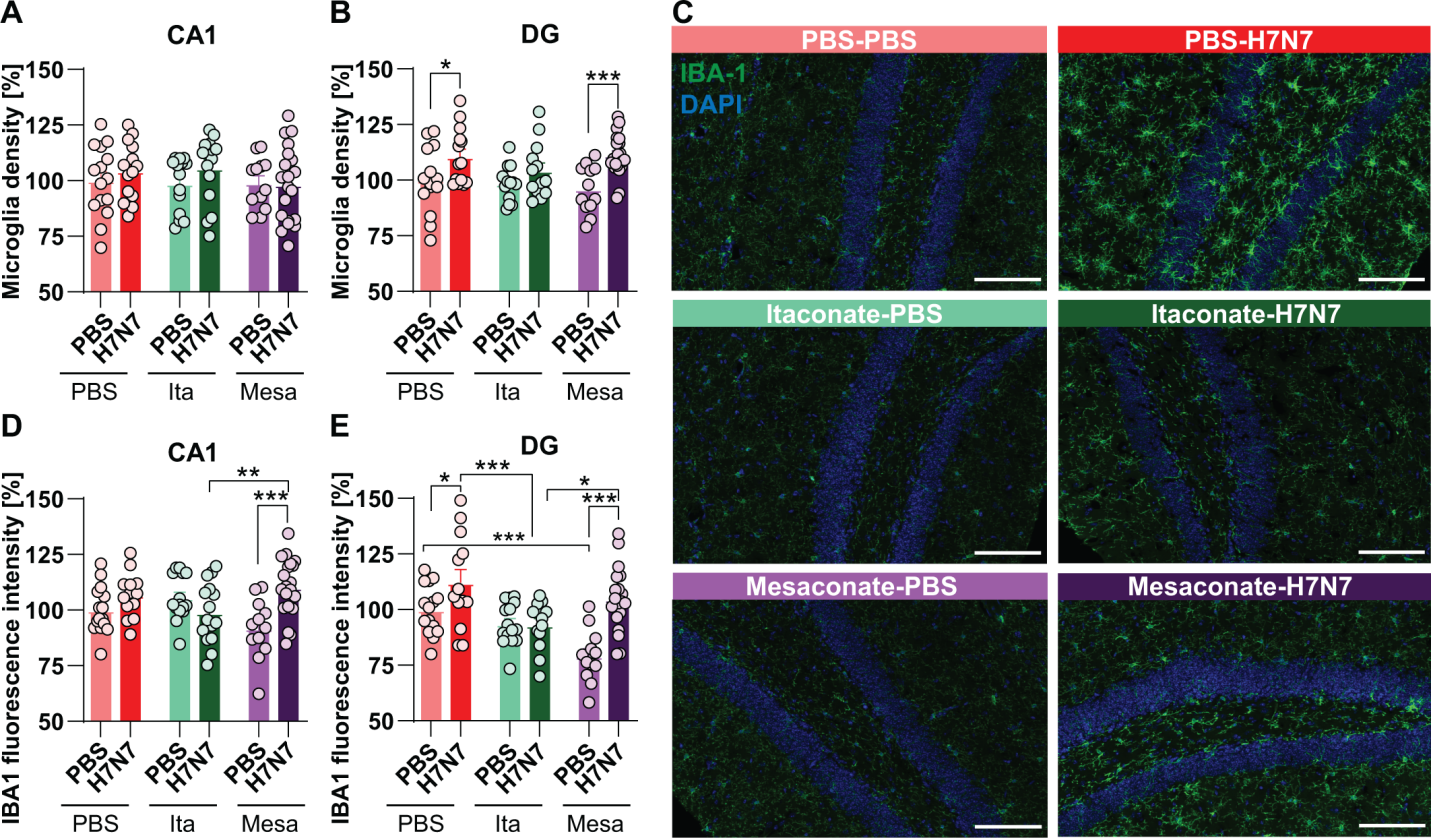
Effects of itaconate and mesaconate on microglial density and activation in the hippocampus following H7N7 infection. Microglial density **(A, B)** and activation **(D, E)** were assessed in the CA1 and dentate gyrus (DG) of PBS-inoculated control and H7N7-infected mice treated with PBS, itaconate, or mesaconate at 8 dpi. **(A, B)** Quantification of IBA-1^+^ microglial density revealed a significant infection-induced increase in the DG, whereas itaconate treatment prevented marked increases in both hippocampal subregions. **(C)** Representative images of IBA-1 (green) immunohistochemical staining with DAPI (blue) nuclear counterstaining in the DG (scale bar, 100 μm). **(D, E)** IBA-1 fluorescence intensity, used as an index of microglial reactivity, was increased following H7N7 infection and was attenuated by itaconate, but not mesaconate, treatment. In contrast, mesaconate treatment was associated with a reduction in IBA-1 fluorescence intensity in non-infected mice. Data are presented as mean ± SEM and analyzed at the level of individual regions of interest (ROIs). Statistical analyses were performed based on the number of ROIs (n) using two-way ANOVA followed by Fisher’s least significant difference (LSD) *post hoc* test (*p < 0.05, **p < 0.01, ***p < 0.001). Group sizes ranged from n = 12–20 IBA-1^+^ imaging frames obtained from N = 3–4 mice per group.

Previous studies have shown that IAV infection induces sustained increases in microglial density and reactivity in the hippocampus during the acute phase of infection and up to 30 days post-infection (7–9, 21). To determine whether treatment with the metabolites itaconate or mesaconate modulates these microglial alterations following H7N7 IAV infection, microglial density and reactivity were assessed in the CA1 and dentate gyrus (DG) subregions of the hippocampus by immunohistochemical analysis of IBA-1-positive cells (**Figure 3**).

At 8 dpi, H7N7-infected mice treated with PBS exhibited a modest, non-significant increase in the number of IBA-1^+^ cells in the CA1 region compared with non-infected control mice (two-way ANOVA, *F*(1, 84) = 1.29, *p* = 0.26; **Figure 3A**). In contrast, a significant increase in microglial density was observed in the DG of PBS-treated infected mice (two-way ANOVA, *F*(1, 80) = 17.71, *p* < 0.0001), with *post hoc* analysis confirming a significant difference between PBS-treated control and infected groups (*p* = 0.01; **Figure 3B**). A similar pattern was observed in mesaconate-treated mice, in which H7N7 infection resulted in a modest increase in microglial density in CA1 and a significant increase in the DG compared with the corresponding control group (PBS-Mesa vs. H7N7-Mesa, *p* = 0.0005; **Figure 3B**). In contrast, itaconate treatment was associated with only slight, non-significant increases in microglial density in both CA1 and DG subregions following infection (**Figures 3A–C**). Direct comparisons among infected and non-infected groups revealed that neither itaconate nor mesaconate treatment significantly altered microglial density relative to PBS-treated mice in either hippocampal subregion (**Figures 3A–C**).

To evaluate microglial reactivity, IBA-1 fluorescence intensity was quantified as an index of microglial activation. Increased IBA-1 fluorescence intensity reflects elevated IBA-1 expression associated with microglial hypertrophy, cytoskeletal remodeling, and enhanced immune reactivity (23, 24) (**Figures 3D, E**). H7N7-infected mice treated with PBS exhibited increased IBA-1 fluorescence intensity in both CA1 and DG compared with control mice (two-way ANOVA, CA1: *F*(1, 84) = 5.74, *p* = 0.01; DG: *F*(1, 81) = 17.73, *p* < 0.001), reaching statistical significance in the DG (PBS-treated control vs. infected, *p* = 0.02; **Figure 3E**). In mesaconate-treated mice, H7N7 infection induced robust increases in IBA-1 fluorescence intensity in both hippocampal subregions relative to their respective controls (CA1: *p* < 0.001; DG: *p* < 0.001; **Figures 3D, E**).

In contrast, itaconate treatment prevented infection-induced increases in microglial reactivity, as no significant differences in IBA-1 fluorescence intensity were detected between infected and control mice in either CA1 or DG (**Figures 3D, E**). Comparative analyses among infected groups revealed that IBA-1 fluorescence intensity in the CA1 region was significantly lower in itaconate-treated mice than in mesaconate-treated mice (*p* = 0.006; **Figure 3D**). In the DG, itaconate-treated infected mice exhibited significantly reduced IBA-1 fluorescence intensity compared with both PBS-treated (*p* = 0.0006) and mesaconate-treated infected mice (*p* = 0.02; **Figures 3D, E**). Notably, comparisons among the non-infected groups revealed that control mice receiving mesaconate exhibited significantly lower IBA-1 fluorescence intensity in the DG compared with PBS-treated control mice (*p* = 0.0001; **Figure 3E**). A plausible explanation is that mesaconate exerts a basal anti-inflammatory or immunomodulatory effect in the brain, dampening homeostatic microglial activation states and thereby reducing IBA-1 expression; however, the strong pro-inflammatory signaling triggered by infection likely overrides these basal immunomodulatory effects, driving robust IBA-1 upregulation that cannot be fully suppressed under pathological conditions.

Collectively, these data indicate that itaconate treatment attenuated H7N7 IAV-associated increases in microglial reactivity in the hippocampus, whereas effects on microglial density were modest and region-specific.

**Figure 4:**
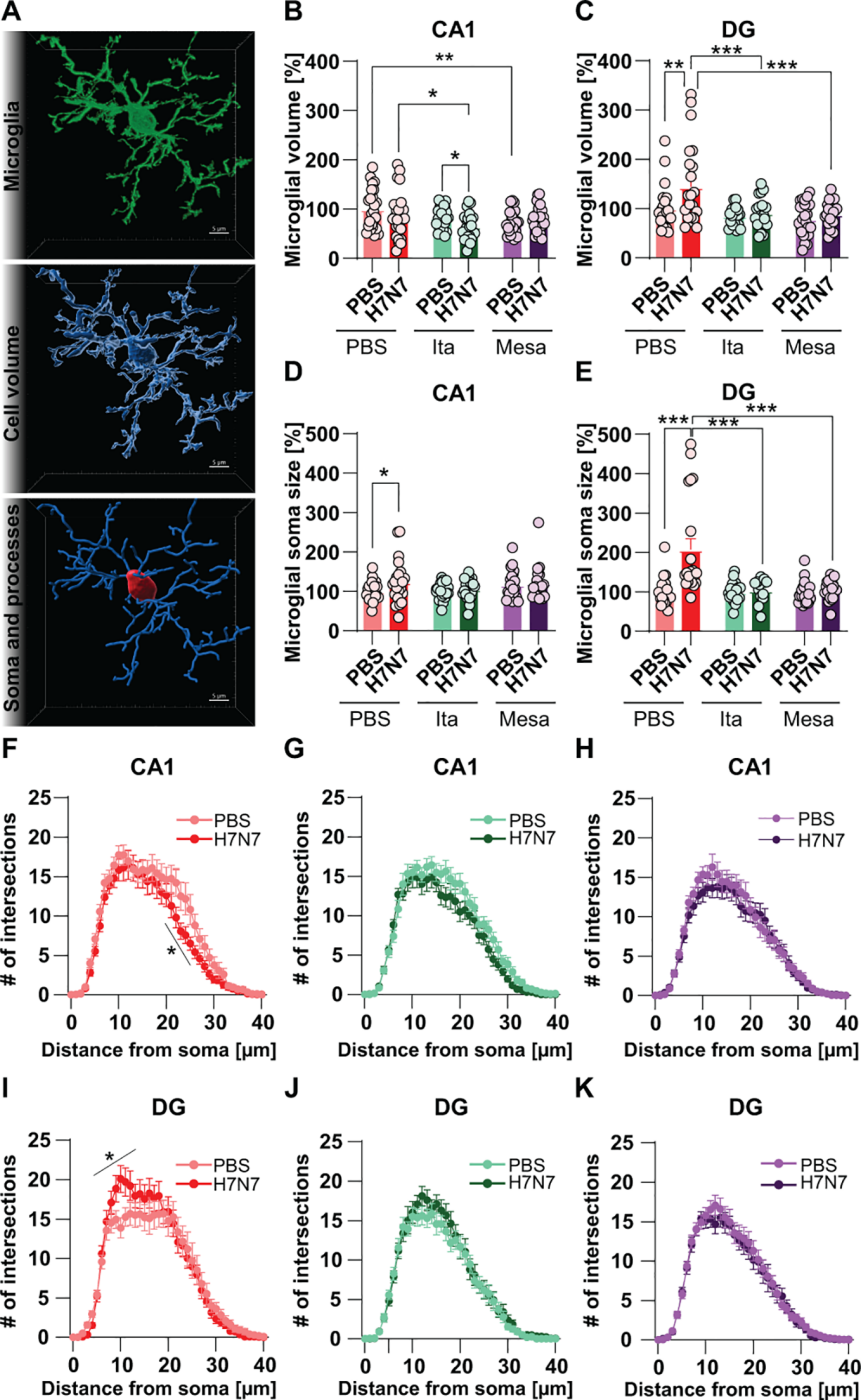
Modulation of hippocampal microglial morphology by H7N7 infection and immunometabolite treatment. **(A)** Representative three-dimensional reconstructions of randomly selected IBA-1^+^ microglia from the CA1 generated using IMARIS^®^ (scale bar, 5 μm). **(B, C)** Quantification of total microglial cell volume in CA1 **(B)** and DG **(C)**. **(D, E)** Quantification of microglial soma volume in CA1 **(D)** and DG **(E)**. **(F–K)** Sholl analysis of microglial process complexity in CA1 **(F–H)** and DG **(I–K)**. H7N7 infection induced region-dependent morphological changes, including increased cell **(B, C)** and soma volume **(D, E)** and altered process complexity **(F–K)**, which were most pronounced in the DG. Treatment with itaconate or mesaconate largely inhibited these infection-induced alterations, indicating attenuation of microglial reactivity. Data are presented as mean ± SEM and analyzed at the level of randomly selected IBA-1^+^ microglia. Statistical analyses were performed based on the number of randomly selected cells **(n)** using two-way ordinary ANOVA or two-way repeated-measures ANOVA (for Sholl analysis), followed by Fisher’s LSD *post hoc* test (*p < 0.05, **p < 0.01, ***p < 0.001). Group sizes ranged from n = 18–26 randomly selected IBA-1^+^ cells obtained from N = 3–4 mice per group.

To further characterize microglial reactivity at the single-cell level, three-dimensional morphological analyses were performed on microglial cells in the CA1 and DG subregions of the hippocampus. Microglial activation is associated with characteristic morphological changes, including alterations in total cell and soma volume as well as changes in process architecture, with microglia exhibiting either hyper-ramification during early or moderate activation states or process retraction and hypertrophy during more severe inflammatory conditions (25, 26). Accordingly, three-dimensional reconstructions of randomly selected IBA-1^+^ microglial cells were generated using IMARIS^®^ software (**Figure 4A**). Quantitative analyses of total cell volume, soma volume, and process complexity were conducted separately for each hippocampal subregion.

Analysis of total microglial cell volume revealed region-specific effects of H7N7 infection. In the CA1 subregion, microglial cell volume did not differ significantly between PBS-treated H7N7-infected mice and PBS-inoculated control mice (two-way ANOVA, *F*(1, 138) = 2.44, *p* = 0.12; **Figure 4B**). In contrast, in the DG, H7N7 infection resulted in a significant increase in microglial cell volume relative to controls (two-way ANOVA, *F*(1, 128) = 6.36, *p* = 0.01), with *post hoc* analysis confirming a significant difference between PBS-treated control and infected groups (*p* = 0.001; **Figure 4C**).

Treatment with itaconate significantly reduced microglial cell volume in the CA1 of infected mice compared with the corresponding itaconate-treated control group (PBS-Ita vs. H7N7-Ita, *p* = 0.04; **Figure 4B**), whereas no significant effect was observed in the DG (**Figure 4C**). Infected mice treated with mesaconate did not exhibit significant differences in microglial cell volume in either hippocampal subregion compared with their respective control groups (**Figures 4B, C**). Notably, direct comparisons among infected groups revealed that microglial cell volume was significantly reduced in itaconate-treated mice compared with PBS-treated infected mice in the CA1 (*p* = 0.01) and was markedly reduced in the DG in mice treated with either itaconate (*p* = 0.0002) or mesaconate (*p* < 0.0001; **Figures 4B, C**).

It is worth noting that comparisons among non-infected groups revealed a significant reduction in microglial cell volume in the hippocampus of control mice treated with mesaconate, with the effect being most pronounced in the CA1 subregion, compared with PBS-treated control mice (*p* = 0.004; **Figure 4B**). As mentioned above, these findings support the notion that mesaconate exerts a basal anti-inflammatory or immunomodulatory effect in the brain, potentially dampening homeostatic microglial activation states.

Next, microglial soma volume was quantified, as reactive microglia are typically characterized by enlarged somata (27). In PBS-treated mice, H7N7 infection induced a significant increase in soma volume relative to control animals, with region-specific effects. In the CA1, *post hoc* analysis revealed a significant difference between PBS-treated control and infected groups (*p* = 0.02), despite the absence of a significant main effect of infection (two-way ANOVA, *F*(1, 126) = 2.64, *p* = 0.10; **Figure 4D**). In contrast, in the DG, H7N7 infection resulted in a robust increase in soma volume (two-way ANOVA, *F*(1, 112) = 13.34, *p* = 0.0004), with *post hoc* analysis confirming a significant difference between PBS-treated control and infected groups (*p* < 0.0001; **Figure 4E**).

Infected mice treated with either itaconate or mesaconate did not exhibit significant increases in soma volume in either hippocampal subregion compared with their respective control groups (**Figures 4D, E**). Moreover, in the DG, soma volume was significantly reduced in infected mice treated with itaconate or mesaconate compared with PBS-treated infected mice (both *p* < 0.0001; **Figure 4E**).

Finally, microglial process complexity was assessed using Sholl analysis (**Figures 4F–K**). In the CA1 subregion, H7N7-infected mice treated with PBS exhibited a significant reduction in microglial process complexity compared to control mice, particularly at distal distances from the soma (two-way repeated-measures ANOVA: *F*(44,1760) = 25.53, *p* < 0.0001; **Figure 4F**). This reduction was not observed in infected mice treated with either itaconate or mesaconate, which displayed process complexity profiles comparable to their respective control groups (**Figures 4G, H**).

In contrast, in the DG, H7N7-infected mice treated with PBS showed a significant increase in microglial process complexity in regions proximal to the soma compared to control mice (two-way repeated-measures ANOVA: *F*(38,1520) = 27.50, *p* < 0.0001; **Figure 4I**). This alteration was not detected in infected mice treated with itaconate or mesaconate, whose process complexity profiles remained similar to those of the corresponding control groups (**Figures 4J, K**).

Collectively, these data demonstrate that H7N7 infection induces pronounced, region-dependent morphological alterations in hippocampal microglia, characterized by increased total cell volume, soma enlargement, and altered process complexity. Treatment with itaconate largely prevented these infection-induced morphological changes, consistent with an attenuation of microglial reactivity in response to IAV infection (**Figure 3**). Notably, mesaconate treatment also modulated microglial morphology to some extent, with potential effects on homeostatic microglial states.

### 3.4 Metabolite treatment modulates H7N7 IAV-induced microglial synaptic remodeling at 8 dpi

**Figure 5:**
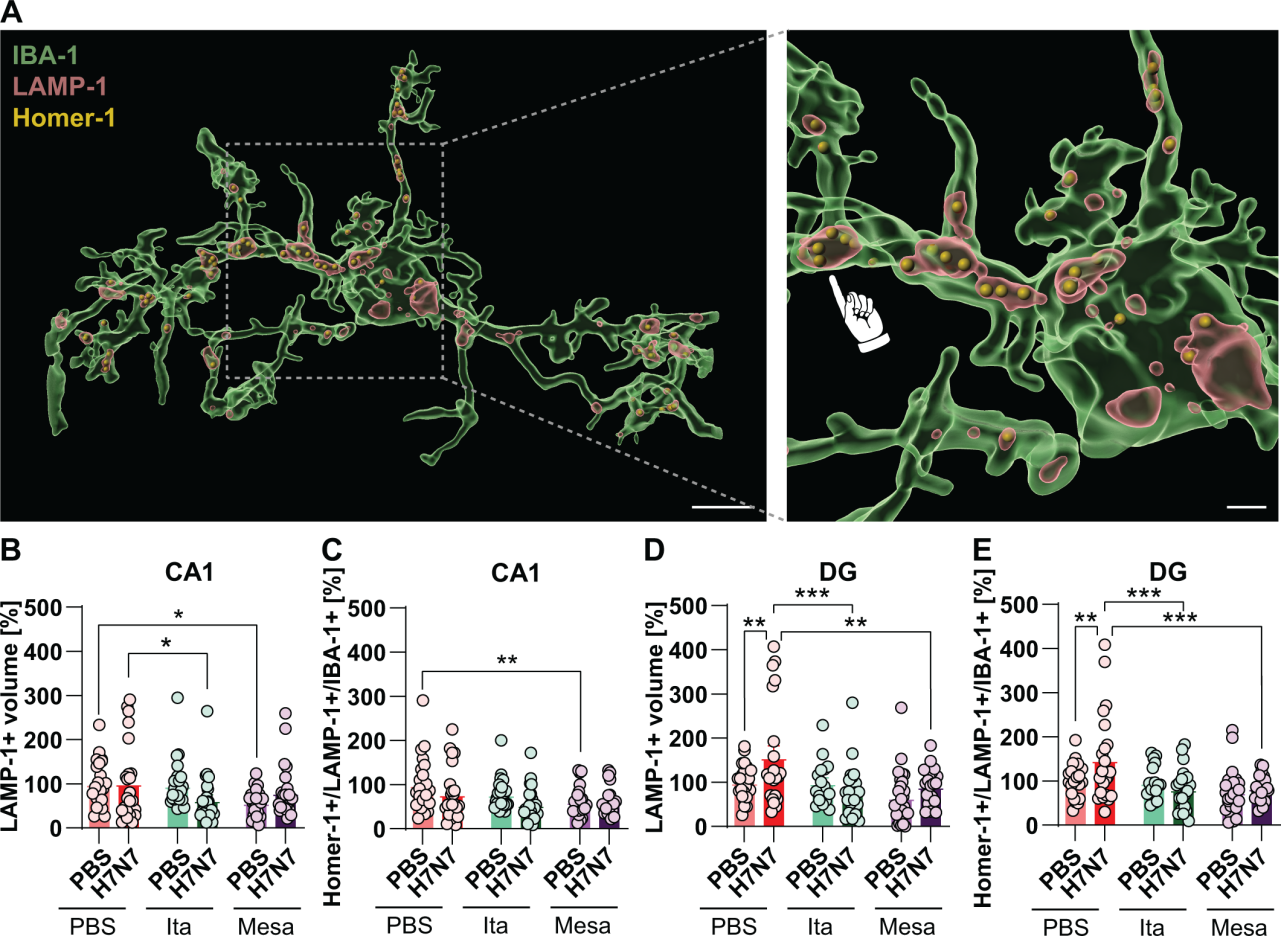
H7N7 infection-induced microglial lysosomal activity and postsynaptic engulfment are regulated by itaconate and mesaconate. **(A)** Representative three-dimensional reconstructions of IBA-1^+^ microglia immunolabeled for the lysosomal marker LAMP-1 and the postsynaptic protein Homer-1 (scale bar, 5 and 2 μm). **(B, D)** Quantification of LAMP-1-positive compartment volume within microglial cells in the CA1 **(B)** and DG **(D)**. **(C, E)** Quantification of Homer-1-positive puncta localized within LAMP-1-positive compartments of microglia in the CA1 **(C)** and DG **(E)**. H7N7 infection was associated with increased microglial lysosomal volume and enhanced localization of postsynaptic material in the DG, effects that were largely prevented by treatment with itaconate or mesaconate. Data are presented as mean ± SEM and analyzed at the level of randomly selected IBA-1^+^ microglia. Statistical analyses were performed based on the number of randomly selected cells **(n)** using two-way ANOVA followed by Fisher’s LSD *post hoc* test (*p < 0.05, **p < 0.01 and ***p < 0.001). Group sizes ranged from n = 18–26 randomly selected IBA-1^+^ cells obtained from N = 3–4 mice per group.

Activated microglia have been reported to be associated with disruptions of brain homeostasis and alterations in synaptic connectivity (28). Previous studies have shown that microglia are capable of engulfing synaptic elements and contribute to synaptic pruning, including during viral infection (21, 29, 30). This process involves lysosome-associated pathways, as lysosomes are key organelles for cellular catabolism and can vary in number, size, and subcellular distribution depending on the functional state of the cell (31). Based on these observations, microglial engulfment of postsynaptic compartments was assessed. To this end, triple immunohistochemical staining for IBA-1, LAMP-1, and Homer-1 was performed. The volume of LAMP-1-positive compartments within IBA-1-positive microglial cells, as well as the number of Homer-1-positive puncta localized within LAMP-1-positive compartments, were quantified in three-dimensionally reconstructed microglia using IMARIS^®^ software (**Figure 5A**).

First, the volume of LAMP-1-positive compartments within microglial cells was quantified (**Figures 5B, D**). In the CA1 subregion, H7N7 infection did not significantly alter microglial LAMP-1 volume in PBS-treated mice compared to controls (two-way ANOVA: *F*(1,137) = 0.07, *p* = 0.78; **Figure 5B**). In contrast, in the DG, H7N7 infection was associated with an increase in LAMP-1 volume relative to control mice, as indicated by *post hoc* analysis (PBS-PBS vs. H7N7-PBS, *p* = 0.004), despite the absence of a significant main effect of infection (two-way ANOVA: *F*(1,128) = 3.48, *p* = 0.06; **Figure 5D**). Notably, this infection-associated increase in LAMP-1 volume was not observed in either hippocampal subregion of H7N7-infected mice treated with itaconate or mesaconate (**Figures 5B, D**). Moreover, infected mice treated with itaconate exhibited a significantly lower LAMP-1 volume compared to PBS-treated infected mice in both the CA1 and DG (H7N7-PBS vs. H7N7-Ita; CA1: *p* = 0.02; DG: *p* = 0.0003; **Figures 5B, D**). In comparison, mesaconate-treated infected mice showed a significant reduction in LAMP-1 volume relative to PBS-treated infected mice only in the DG (H7N7-PBS vs. H7N7-Mesa, *p* = 0.001; **Figure 5D**). In the CA1 subregion, mesaconate treatment in non-infected mice resulted in a significant reduction in LAMP-1 volume compared with PBS-treated control mice, further indicating that certain homeostatic features of microglia can be modulated by this metabolite (*p* = 0.0015; **Figure 5A**).

Subsequently, to assess synaptic engulfment by microglia, the number of Homer-1-positive puncta localized within lysosomal compartments of individual microglial cells was quantified across experimental groups (**Figures 5C, E**). Homer-1 is a postsynaptic scaffolding protein that regulates glutamatergic synapses and dendritic spine architecture. Reduced Homer-1 levels are associated with impaired glutamatergic signaling, altered spine morphology, and decreased synaptic stability (32).

In the CA1 subregion, H7N7 infection did not significantly alter the number of Homer-1-positive puncta within LAMP-1-positive compartments of microglial cells in either PBS- or metabolite-treated mice compared with controls (two-way ANOVA: *F*(1,133) = 3.46, *p* = 0.06; **Figure 5C**). However, in the non-infected group, mesaconate treatment resulted in a significant reduction in Homer-1-positive puncta within LAMP-1-positive compartments of microglial cells compared with PBS-treated control mice, which may again reflect modulation of microglial phagocytic activity under homeostatic conditions (*p* = 0.005; **Figure 5C**).

In contrast, in the DG, H7N7-infected mice treated with PBS exhibited a significant increase in the number of Homer-1-positive puncta localized within microglial lysosomes compared to control animals, as revealed by *post hoc* analysis (PBS-PBS vs. H7N7-PBS, *p* = 0.004), despite the absence of a significant main effect of infection (two-way ANOVA: *F*(1,126) = 2.06, *p* = 0.15; **Figure 5E**).

This infection-associated increase was not observed in either hippocampal subregion of H7N7-infected mice treated with itaconate or mesaconate. Notably, in the DG, infected mice treated with either metabolite exhibited a significantly lower number of Homer-1-positive puncta within lysosomal compartments compared to PBS-treated infected mice (H7N7-PBS vs. H7N7-Ita, *p* = 0.0002; H7N7-PBS vs. H7N7-Mesa, *p* = 0.0002; **Figure 5E**).

Taken together, these results indicate that H7N7 infection is associated with increased localization of postsynaptic material within microglial lysosomes in the DG, an effect that is largely absent in infected mice treated with metabolites.

### 3.5 H7N7 IAV-induced impairments in synaptic plasticity observed at 30 dpi were absent in itaconate-treated mice

We previously demonstrated that H7N7 IAV infection induces persistent alterations in hippocampal neuronal function, evidenced by a significant reduction in long-term potentiation (LTP) in acute hippocampal slices obtained from infected mice at 30 dpi (7). Because metabolite treatment – particularly itaconate – modulated microglial morphology associated with activation during the acute phase of infection, we next asked whether such treatment could prevent the long-term synaptic plasticity deficits induced by H7N7 infection.

**Figure 6:**
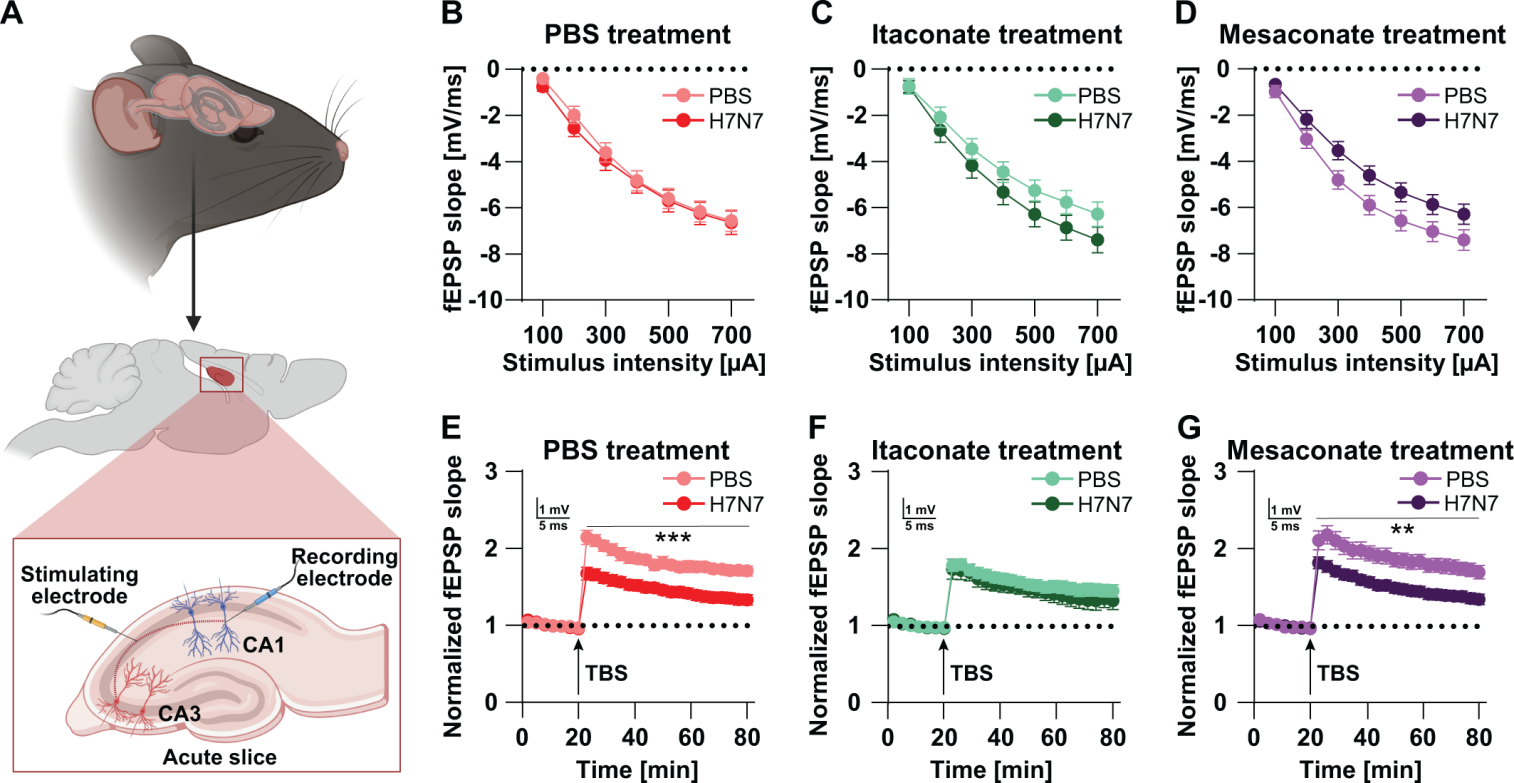
Effects of H7N7 infection and immunometabolite treatment on hippocampal synaptic plasticity at 30 dpi. **(A)** Schematic of field potential recordings at the Schaffer collateral CA3-CA1 synapse in acute hippocampal slices (*Created in BioRender*). **(B–D)** Input-output curves showing basal synaptic transmission in PBS- **(B)**, itaconate- **(C)**, and mesaconate-treated **(D)** mice. H7N7 infection did not significantly affect basal synaptic transmission in any treatment group. **(E–G)** Long-term potentiation (LTP) induced by theta-burst stimulation at the CA3-CA1 synapse in PBS- **(E)**, itaconate- **(F)**, and mesaconate-treated **(G)** mice. H7N7 infection significantly reduced LTP magnitude in PBS- and mesaconate-treated mice, whereas LTP was preserved in infected mice treated with itaconate. Data are presented as mean ± SEM and analyzed at the level of acute hippocampal slices. Statistical analyses were performed based on the number of acute hippocampal slices (n) using two-way repeated-measures ANOVA, followed by Fisher’s LSD *post hoc* test (**p < 0.01 and ***p < 0.001). Group sizes ranged from n = 15–25 acute hippocampal slices obtained from N = 3–5 mice per group.

To address this question, synaptic plasticity was assessed at the Schaffer collateral pathway connecting the CA3 and CA1 hippocampal subfields, one of the most extensively characterized synapses in the CNS, using acute hippocampal slices from all experimental groups (**Figure 6A**). Basal synaptic transmission was first evaluated by examining the relationship between stimulation intensity and the slope of the field excitatory postsynaptic potential (fEPSP) using input-output curves (**Figures 6B–D**). No significant effects of infection were detected in mice treated with PBS, itaconate, or mesaconate compared with their respective control groups (two-way repeated-measures ANOVA: PBS, *F*(1,43) = 0.16, *p* = 0.68, **Figure 6B**; itaconate, *F*(1,33) = 1.41, *p* = 0.24, **Figure 6C**; mesaconate, *F*(1,43) = 3.89, *p* = 0.054, **Figure 6D**). These results indicate that H7N7 infection did not induce persistent alterations in basal synaptic transmission. Although mesaconate-treated H7N7-infected mice exhibited a modest trend toward reduced fEPSP slope, this effect did not reach statistical significance and likely reflects subtle modulation of synaptic gain rather than a persistent disruption of basal excitatory transmission.

Long-term synaptic plasticity was subsequently examined (**Figures 6E–G**). LTP at the Schaffer collateral CA3-CA1 synapse was induced using theta-burst stimulation (TBS) following 20 minutes of stable baseline recording. Consistent with our previous findings (7), H7N7-infected mice treated with PBS exhibited a significant reduction in LTP magnitude compared with control animals at 30 dpi (two-way repeated-measures ANOVA: *F*(1,43) = 15.23, *p* = 0.0003; **Figure 6E**). A comparable impairment in LTP was observed in H7N7-infected mice treated with mesaconate (*F*(1,43) = 11.04, *p* = 0.0018; **Figure 6G**). In contrast, LTP magnitude in H7N7-infected mice treated with itaconate did not differ significantly from that observed in non-infected itaconate-treated controls (*F*(1,33) = 0.50, *p* = 0.48; **Figure 6F**).

Notably, although itaconate treatment alone was associated with a reduction in LTP magnitude relative to PBS-treated controls – potentially reflecting a shift toward synaptic stabilization or altered plasticity thresholds under homeostatic conditions – H7N7 infection did not result in a significant impairment of LTP in itaconate-treated animals. These findings suggest that itaconate-mediated attenuation of neuroinflammatory signaling during the acute phase of H7N7 infection may contribute to the preservation of synaptic plasticity, potentially involving modulation of microglial activation as well as effects on other neural or immune cell populations and/or viral dynamics within the brain.

## 4 Discussion

Building on previous evidence demonstrating robust anti-inflammatory and neuroprotective effects of itaconate and mesaconate in models of LPS-induced neuroinflammation – including reduced microglial activation, attenuated cytokine production, and preservation of synaptic plasticity (19) – we sought to determine whether similar immunometabolic modulation could mitigate the neurological sequelae of H7N7 IAV infection. Respiratory IAV infections are increasingly recognized for their capacity to induce secondary neuroinflammation and long-lasting cognitive impairments, even in the absence of pronounced neurotropism for certain strains (7, 9, 10, 21). In this context, the present study demonstrates that therapeutic modulation of immunometabolism during acute H7N7 IAV infection differentially affects systemic sickness responses, neuroinflammation, microglial activation, synaptic remodeling, and long-term hippocampal synaptic plasticity.

In the present study, a dose of 250 mg/kg was selected based on prior *in vivo* studies demonstrating anti-inflammatory effects of itaconate and mesaconate (18, 19). Endogenously, itaconate accumulates to millimolar concentrations in activated macrophages, whereas mesaconate is present at approximately tenfold lower levels but exhibits comparable cytotoxicity and anti-inflammatory potency (18, 33). Compared with cell-permeable itaconate derivatives, the natural forms of both metabolites display substantially lower cytotoxicity and milder anti-inflammatory activity. *In vivo*, itaconate is rapidly cleared and considered a short-lived metabolite (34); although pharmacokinetic data for mesaconate remain limited, its structural similarity to itaconate and comparable cellular effects support similar dosing and administration strategies. Collectively, these considerations – including low cytotoxicity, moderate anti-inflammatory efficacy, rapid clearance, and relatively limited cellular uptake – guided the selection of the dosing regimen used in this study.

While neither itaconate nor mesaconate substantially altered overall disease severity, as assessed by body weight loss, mesaconate selectively attenuated infection-associated hypothermia during the peak of disease. These findings align with previous reports showing that influenza infection induces pronounced sickness behavior, including hypothermia and weight loss, reflecting robust systemic inflammatory and metabolic responses (7, 21, 35). The absence of an effect on body weight loss suggests that, under the applied dosing regimen, immunometabolic intervention does not substantially alter the overall course of acute IAV disease. In contrast, the partial normalization of body temperature observed following mesaconate treatment indicates a selective influence on sickness-associated physiological regulation, which is closely linked to inflammatory and metabolic signaling pathways during infection (15, 18). However, the absence of viral load and tissue tropism analyses in the present study precludes definitive conclusions regarding the effects of itaconate and mesaconate on viral replication or antiviral host defense. Nevertheless, previous *in vitro* studies have demonstrated that, in a human monocyte-like cell line infected with the H1N1 IAV strain PR8M, both itaconate and mesaconate profoundly alter amino acid metabolism, modulate cytokine/chemokine release, and attenuate interferon signaling, oxidative stress, and viral particle release (33).

Consistent with previous reports, H7N7 infection induced elevated levels of pro-inflammatory cytokines in both lung and brain tissue, supporting the concept that respiratory viral infections – either through direct replication within brain cells or indirectly via peripheral immune signaling – can elicit secondary neuroinflammatory responses (7, 21, 36). Neither itaconate nor mesaconate reduced pulmonary cytokine production at 8 dpi, indicating that systemic antiviral inflammatory responses remained largely intact. In the brain, however, metabolite-specific effects emerged, as itaconate treatment was associated with increased concentrations of TNF-α, IL-6, and IL-1β compared with infected PBS- or mesaconate-treated mice. The inflammatory responses observed following metabolite treatment in the lung and brain may reflect an augmented host immune response, which could contribute to improved control of viral replication; however, this possibility requires further investigation. These findings contrast with previous studies demonstrating anti-inflammatory effects of itaconate in endotoxin-driven neuroinflammation, where it suppresses NF-κB signaling, inflammasome activation, and oxidative stress (37–39). This discrepancy likely reflects context-dependent effects of itaconate, as its immunomodulatory properties are influenced by dose, timing, and the nature of the inflammatory stimulus. Indeed, recent work has shown that electrophilic and Nrf2-activating metabolites can exert anti-inflammatory effects at lower exposures while promoting IL-1β production or cellular stress responses under certain conditions (40). In contrast to itaconate, mesaconate selectively reduced brain IL-1β levels, a cytokine strongly implicated in infection-induced synaptic dysfunction and cognitive impairment, suggesting a modest but potentially relevant neuroprotective effect during the acute phase of disease (41).

At the cellular level, H7N7 infection induced region-specific microglial activation within the hippocampus, with the dentate gyrus showing greater susceptibility than CA1. This pattern is consistent with earlier studies reporting sustained microglial activation and neuronal alterations in the hippocampus following influenza infection (7, 21). The heightened vulnerability of the DG may be related to its unique neurogenic niche, as it is one of the few regions in the adult mammalian brain that harbors neural stem and progenitor cells. Adult hippocampal neurogenesis is highly sensitive to inflammatory cues, and both viral infection and microglial activation have been shown to profoundly disrupt progenitor cell survival, differentiation, and synaptic integration (42, 43). Moreover, neurotropic and non-neurotropic viruses have been reported to preferentially affect neurogenic regions, potentially due to the heightened metabolic activity and plasticity of neural stem cells, rendering the DG a particularly vulnerable target during infection (30, 44, 45).

Microglial activation is a multidimensional process that cannot be adequately described by a single parameter. In the present study, we therefore employed complementary readouts, including IBA-1-based morphological analyses and measures of synaptic engulfment, which reflect distinct but related aspects of microglial functional state. Whereas morphological changes provide a sensitive indicator of activation, synaptic remodeling represents a downstream, functionally relevant consequence with direct implications for neuronal circuit integrity. Together, these measures offer an integrated assessment of microglia-driven neuropathology during neurotropic IAV infection.

Although changes in microglial density were relatively modest, itaconate effectively prevented infection-induced microglial reactivity, as evidenced by reduced IBA-1 fluorescence intensity and normalization of microglial morphology. Three-dimensional analyses revealed that H7N7 infection induced classical features of microglial activation, including increased total cell and soma volume as well as pronounced alterations in process complexity, manifesting as either reduced or hyper-ramification depending on the hippocampal subregion. These region-dependent morphological patterns likely reflect intrinsic microglial heterogeneity and distinct functional states across hippocampal circuits (46). Notably, these infection-induced morphological changes were largely absent in metabolite-treated mice. Moreover, mesaconate modulated several microglial morphological parameters even in the absence of infection, indicating potential effects on homeostatic microglial functions. Together, these findings support the notion that microglial activation is not solely reflected by changes in cell number and that morphological parameters provide sensitive indicators of functional reprogramming (47). Importantly, the dissociation between cytokine levels and microglial morphology suggests that immunometabolic modulation can directly shape microglial behavior independently of bulk tissue inflammatory mediator concentrations.

A key mechanistic insight from this study is that H7N7 infection promoted microglial engulfment of postsynaptic compartments selectively in the DG, as indicated by increased localization of the postsynaptic scaffold protein Homer-1 within LAMP-1-positive lysosomal compartments. Microglia-mediated synaptic pruning has emerged as a central mechanism underlying cognitive impairment in viral and other neuroinflammatory conditions. In models of neurotropic viral infection, complement-dependent microglial synapse elimination drives memory deficits (29), and during recovery from flavivirus infection, T cell-microglia interactions promote synaptic loss and cognitive dysfunction (30). In the context of influenza, microglial engulfment of synaptic elements has recently been linked to hippocampal neuronal damage and reduced dendritic spine density (21, 36). Homer proteins are essential components of the postsynaptic density and play a critical role in stabilizing glutamatergic synapses and supporting synaptic plasticity (48). The observed increase in Homer-1 within microglial lysosomes therefore likely reflects pathological synaptic remodeling during acute neuroinflammation. Notably, both itaconate and mesaconate markedly reduced microglial lysosomal volume and synaptic engulfment, indicating that immunometabolic modulation restrains microglial phagolysosomal engagement with synaptic structures and may represent a promising strategy to limit influenza-associated neurological sequelae. However, the direct mechanisms underlying these effects remain to be identified.

Functionally, these early cellular effects were associated with long-term consequences for synaptic plasticity. In line with previous work, H7N7 infection induced persistent impairments in hippocampal long-term potentiation (LTP) at 30 dpi (7, 21). These findings support the notion that acute infection-induced neuroinflammation contributes to long-lasting synaptic dysfunction. Notably, the LTP deficit was absent in itaconate-treated mice at 30 dpi, whereas mesaconate-treated mice exhibited LTP impairments comparable to those observed in PBS-treated infected animals. These findings indicate that early suppression of microglial expansion, activation, and synaptic engulfment by itaconate during the acute phase of infection is sufficient to prevent the development of long-term synaptic dysfunction.

Interestingly, itaconate treatment alone reduced LTP magnitude in control mice, indicating that basal immunometabolic modulation can influence synaptic plasticity under physiological conditions. Nevertheless, the absence of additional LTP impairment following H7N7 infection implies that itaconate confers resilience against infection-induced pathological synaptic remodeling. In contrast, mesaconate failed to confer comparable long-term protection of synaptic plasticity despite attenuating IL-1β and IL-6 levels and partially reducing activation-associated microglial morphological features. Notably, mesaconate did not prevent infection-induced increases in microglial density or IBA-1 expression, suggesting that the persistence of elevated microglial numbers may contribute to synaptic plasticity impairments even when select inflammatory and morphological parameters are normalized. These data collectively imply that effective long-term preservation of synaptic function requires not only dampening microglial inflammatory signaling but also limiting microglial accumulation and sustained engagement with synaptic elements during infection. Consistent with accumulating evidence, these observations support the concept that microglial-synapse interactions during acute inflammatory insults critically shape long-term circuit stability and neuronal function (29, 30).

**Figure 7:**
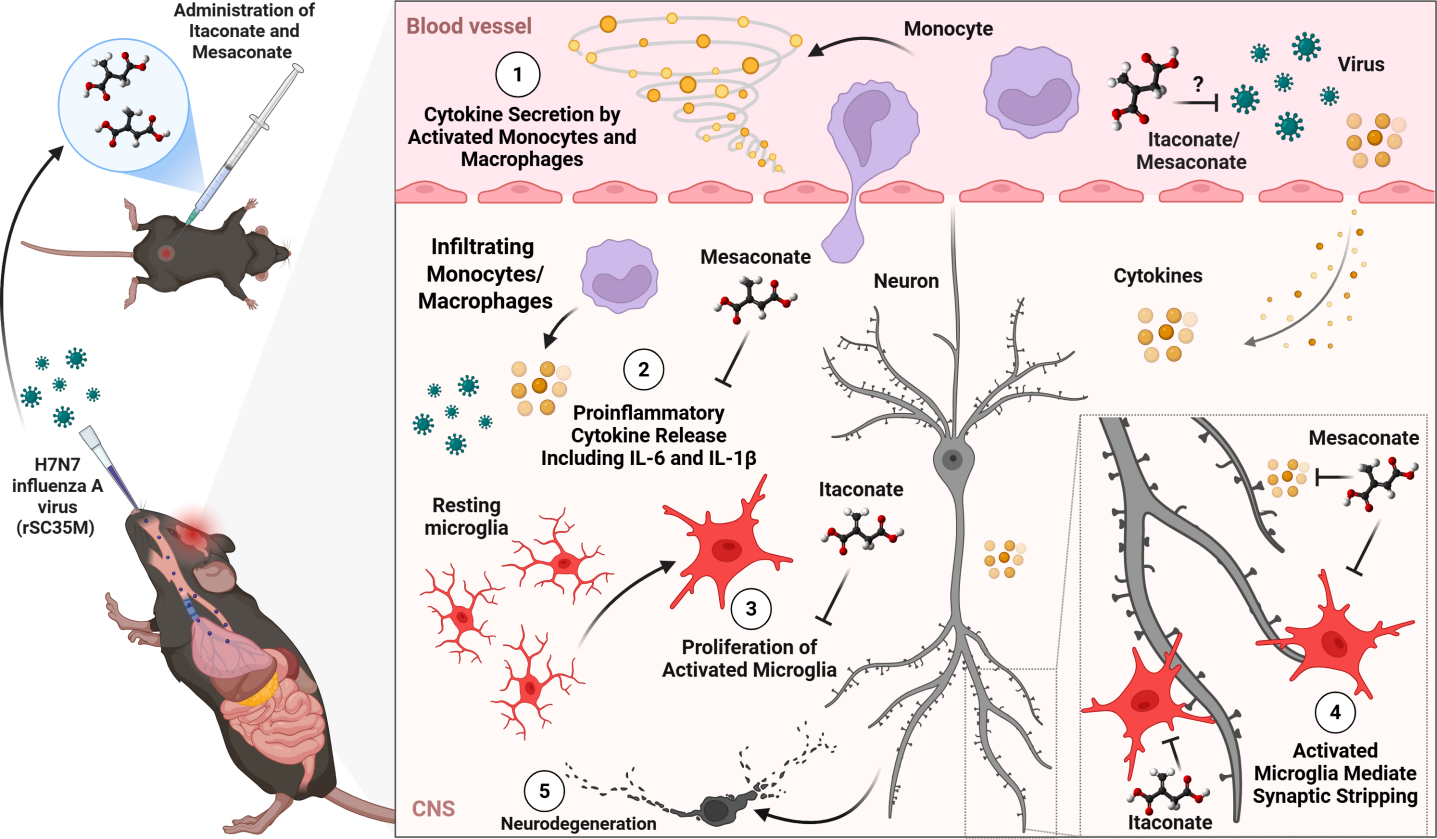
Proposed model of immunometabolic modulation during neurotropic IAV infection. The schematic illustrates that following IAV infection, (1) pro-inflammatory cytokines released by activated peripheral monocytes and macrophages and/or viral particles themselves can access the brain and trigger neuroinflammatory responses. Whether *in vivo* treatment with itaconate or mesaconate directly inhibits viral replication remains unknown. (2) Mesaconate selectively reduced brain IL-1β and IL-6 levels, whereas itaconate (3) more robustly suppressed infection-induced microglial density and activation and attenuated (4) microglial morphological reactivity and synaptic remodeling associated with infection, (5) processes that are implicated in subsequent neurodegenerative changes. Collectively, the model supports emerging evidence that structurally related itaconate isomers engage partially overlapping yet mechanistically distinct signaling programs rather than differing solely in inhibitory potency (*Created in BioRender.**https://BioRender.com/pqfhoem**, is licensed under CC BY 4.0*).

In the present study, itaconate and mesaconate exhibited divergent effects despite their shared immunometabolic origin, underscoring mechanistic differences in the magnitude, duration, or nature of the immunometabolic reprogramming induced by these metabolites (38, 39). Although both compounds display anti-inflammatory properties, particularly in models of LPS-induced inflammation (18, 19), they differ substantially in their metabolic actions. Itaconate directly modulates cellular metabolism through inhibition of succinate dehydrogenase, thereby impacting the tricarboxylic acid cycle and oxidative phosphorylation, whereas mesaconate exerts minimal effects on these pathways. Consistent with this distinction, our data demonstrate overlapping yet non-identical outcomes during neurotropic IAV infection: mesaconate selectively reduced brain IL-1β and IL-6 levels, whereas itaconate more robustly suppressed infection-induced microglial density and activation, attenuated microglial morphological reactivity, and prevented long-term synaptic plasticity impairments. These findings suggest that itaconate confers broader regulation of microglial functional states and synaptic integrity, while mesaconate modulates more discrete inflammatory pathways (**Figure 7**). Collectively, these results support emerging evidence that structurally related itaconate isomers engage partially overlapping but mechanistically distinct signaling programs rather than differing solely in inhibitory potency, warranting further investigation.

Several limitations of this study should be acknowledged. Cytokine measurements were obtained at a single acute time point, limiting insight into the temporal dynamics of the inflammatory response; longitudinal profiling across both acute and recovery phases would therefore be informative. Moreover, cytokine analyses were performed using whole-hemisphere homogenates following systemic administration of itaconate and mesaconate, which precludes definitive conclusions regarding cell type- or region-specific mechanisms within the CNS. Although this approach provides a broad overview of the inflammatory state of the brain, it may mask localized inflammatory changes, particularly within the hippocampus, underscoring the need for future studies employing region-specific tissue analyses and cell type-targeted approaches to more precisely link cytokine signaling to hippocampal pathology. Behavioral assessments were not included, precluding direct correlation of synaptic plasticity alterations with cognitive outcomes. Moreover, only a single viral strain and dosing regimen were examined. Future studies incorporating alternative infection models, treatment schedules, region-specific analyses of viral replication and propagation, and additional synaptic or complement pathway markers would further strengthen mechanistic interpretation.

Although ACOD1/IRG1 expression and endogenous itaconate or mesaconate levels within the brain and specific cell types were not assessed, systemic administration of itaconate and mesaconate markedly attenuated neuroinflammatory signaling and synaptic alterations following IAV infection. These findings suggest that endogenous itaconate responses within the CNS, if present, may be insufficient in magnitude or spatial distribution to fully counteract IAV-induced pathology, and that exogenous supplementation can pharmacologically augment immunometabolic pathways. Future studies examining region- and cell type-specific ACOD1/IRG1 expression will be important to further define the interplay between endogenous and therapeutic itaconate signaling during viral neuroinflammation.

In conclusion, this study identifies microglial immunometabolism as a critical regulator of virus-induced synaptic remodeling and long-term hippocampal dysfunction. The divergent effects of metabolites on neuroinflammatory signaling and synaptic plasticity underscore the complexity of immunometabolic interventions. Targeting microglial metabolic pathways during acute viral infection may therefore represent a promising strategy to limit persistent neurological sequelae following influenza infection.

## Data Availability

The original contributions presented in the study are included in the article/supplementary material. Further inquiries can be directed to the corresponding author.
